# Corticosteroid in anti-inflammatory treatment of pediatric acute myocarditis: a systematic review and meta-analysis

**DOI:** 10.1186/s13052-023-01423-w

**Published:** 2023-03-13

**Authors:** Qi Yao, Shanshan Zhan

**Affiliations:** 1 Department of Cardiology, The First Hospital of Jiaxing / Affiliated Hospital of Jiaxing University, Jiaxing, Zhejiang China; 2 Department of Pediatrics, The First Hospital of Jiaxing / Affiliated Hospital of Jiaxing University, Jiaxing, Zhejiang China

**Keywords:** Acute myocarditis, Corticosteroids, Pediatrics, Left ventricular function

## Abstract

**Background:**

To evaluate the efficacy of corticosteroids in anti-inflammatory treatment of pediatric acute myocarditis.

**Methods:**

We searched PubMed, Embase and Cochrane library and included studies before October 2022 for clinical trials, observational studies and retrospective studies which reported on children with acute myocarditis treated with corticosteroid anti-inflammatory therapy. The quality of the clinical trials was assessed by Jadad score as an exclusion criterion.

**Results:**

This systematic review included 6 studies involving 604 pediatric patients with acute myocarditis. Corticosteroid therapy was not associated with reduced risk of mortality due to acute myocarditis (*P* = 0.53; RR = 0.87; 95% CI = 0.58 to 1.33) compared to anti-failure treatment. There was a significant improvement in pediatric patients’ left ventricular function measured by left ventricular ejection fraction in the group on corticosteroid anti-inflammatory treatment (*P* = 0.0009; MD = 11.93%; 95% CI = 4.87% to 18.99%). No conclusion can be drawn due to the high heterogeneity in meta-analyses of risk of getting to a clinical endpoint (death or heart transplantation) and changes in left ventricular end-diastolic diameter (LVEDD).

**Conclusions:**

Corticosteroid anti-inflammatory therapy in pediatric acute myocarditis patients showed no significant improvement in reducing the risk of mortality, but showed significant improvement in LVEF.

## Background

Myocarditis, an inflammatory disease of the myocardium, remains a clinical challenge in pediatrics as it challenges practitioners in all aspects including diagnosis, methods of intervention, and follow-up counseling [1]. It is a not rare and heterogenous disease in the pediatric age group in both developing and developed countries [2], as a study indicated the evidence of myocarditis in 12% of the autopsied adolescent and young adult patients with sudden death [3]. Myocarditis can cause serious long-term morbidity in children including diminished cardiac function, cardiac failure and potentially necessitating aggressive circulatory support [4]. It can also lead to the development of a chronic dilated cardiomyopathy (DCM), which is a common indication for cardiac transplantation in children older than 1 year [5]. Though patients of all ages may be affected by myocarditis, teenagers and infants, especially children in the first year of life, account for the majority of cases [6]. A recent review on the epidemiology of myocarditis addressed this bimodal age distribution of myocarditis patients without any conclusive reasons [7].

One other characteristic feature of pediatric myocarditis is the multiple etiologies, including viral, immune-mediated, and toxin-mediated [8]. However, the direct pathogen leading to myocarditis in many patients may remain undetermined or mistakenly assigned to viruses, especially PVD19 and HHV6 [5, 9]. At the same time, the diagnosis of myocarditis can be challenging due to the wide spectrum of clinical signs and symptoms [10]. Though the majority of patients with myocarditis would present to their emergency department with a recent previous illness, some patients may have no history of illness before development of symptoms ranging from chest pain, respiratory symptoms, to cardiogenic shock and sudden death [4, 11, 12].

Even though the understanding of pathogenesis in acute myocarditis has been constantly improving, treatments for myocarditis remain controversial and generally focus on supportive care with attention to guideline-directed treatment of heart failure and arrhythmia [10, 13]. Therapies directed towards modulating the immune responses in patients have been considered beneficial in order to antagonize the autoimmune injury to myocytes which exacerbates myocardial dysfunction [14]. Immunosuppressive therapies using steroids in pediatric patients have been conducted in observational and controlled clinical trials but led to confronting conclusions [15–17]. Similar outcomes were also observed in trials conducted on the adult population [18–20].

Previous systematic reviews failed to conclude clinically significant improvements upon administration of immunosuppressive agents against acute myocarditis, largely due to the small number of subjects, especially pediatric studies [17, 21–23]. At the same time, most of the included studies also focused on adult patients. Chen et al. [23] included 8 studies. However only 3 out of those 8 focused on children. The only previous systematic review focusing on the effects of corticosteroids on children with acute myocarditis was done 19 years ago by Hia et al. [17]. At this point, more observational and controlled trials have been conducted on pediatric patients, which provided us with more evidence on revising the effects of immunosuppression in children with myocarditis. Therefore, the objective of this systematic review and meta-analysis was to provide an updated evaluation of the effects of immunosuppression therapies on the outcome of acute myocarditis.

## Material and methods

### Data sources and literature search

In this systematic review and meta-analysis of randomized controlled trials, we performed a systematic literature search with no date limits using PubMed, Embase and Cochrane library without a language limitation. The search was performed to October 1, 2022. The search was performed with the following MeSH terms: “Myocarditis”, “Anti-Inflammatory Agents”, “Immunosuppressive Agents”, “Glucocorticoids” and “Adrenal Cortex Hormones”; and key words: “carditis”, “acute viral myocarditis”, “corticosteroid”, “steroid”, “prednisone”, “dexamethasone”, “hydrocortisone”, “methylprednisone”, “betamethasone”, “budesonide”, “fludrocortisone” and “mineralcorticoids”. The search strategy was as follows: (“Myocarditis” OR “carditis” OR “acute viral myocarditis”) AND (“Anti-Inflammatory Agents” OR “Immunosuppressive Agents” OR “Glucocorticoids” OR “corticosteroid” OR “steroid” OR “prednisone” OR “dexamethasone” OR “hydrocortisone” OR “methylprednisone” OR “betamethasone” OR “budesonide” OR “fludrocortisone” OR “mineralcorticoids”). In addition, two reviewers independently performed manual search based on references from these articles and other review articles.

### Inclusion criteria

The criteria used in the selection of studies for inclusion in this systematic review were as follows: (1) randomized or quasi-randomized controlled trials (RCTs or quasi-RCTs), observational studies, or retrospective studies; (2) human studies; (3) trials included children no more than 18 years old; (4) studies consisting of a minimum of 2 arms, one arm receiving at least one type of corticosteroids and at least one only conventional anti-failure medication.

### Data extraction

Both authors independently performed the literature research and evaluation of the retrieved data. After data extraction and collection, we resolved discrepancy in exclusion criteria by discussion based on literatures available in the database. The studies included by one author were examined by the other author. Quality of the clinical trials were assessed with Jadad score [24].

The information extracted from each trial included: authors, year of publication, sample size, study duration (only for RCTs), mean age or age range, intervention, dosage of intervention, comparator, and dosage of comparator.

### Assessment of methodological quality

We followed the guideline given in Cochrane Handbook for Systematic Reviews of Interventions [25]. The likely magnitude and direction of the bias and whether these biases impacted the findings according to the criteria. The two authors (QY and SZ) independently assessed the quality of studies without blinding to authorship or journal. We resolved discrepancy by discussion.

### Statistical analysis

All statistical analyses were conducted using Review Manager 5.4 statistical software. Dichotomous variables were analyzed using risk ratios (RRs) with 95% confidential interval (95% CI) based on the fixed effects model. Continuous variables were analyzed using mean difference (MD) with 95% CI. Statistical heterogeneity of the included trials was assessed by the *I*^2^ statistics. The random effect model was applied when compared trials had high heterogeneity (*I*^2^ > 50%). We assessed publication bias of included studies using Egger test [26].

## Results

### Literature search

**Fig. 1:**
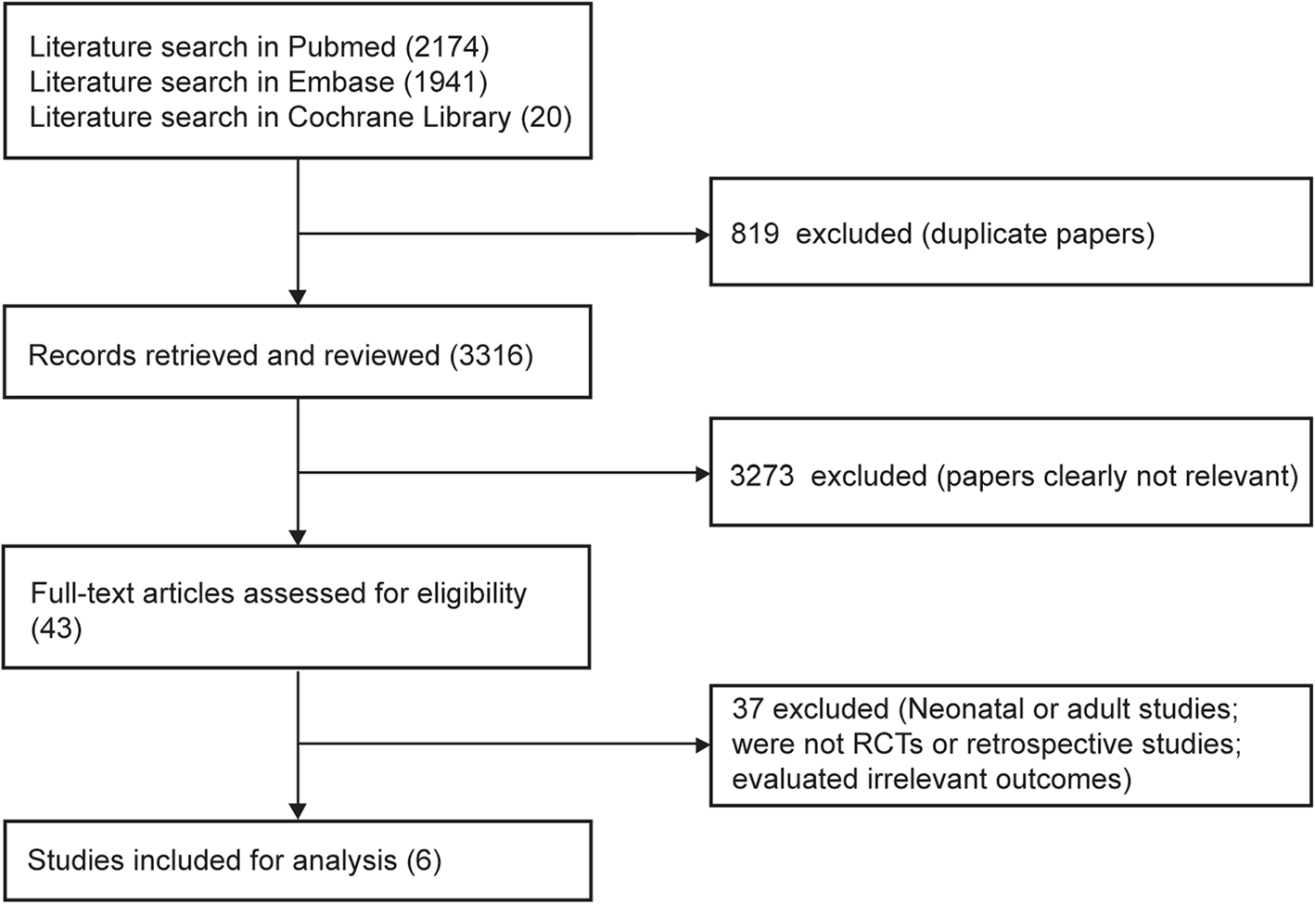
Detailed flowchart of studies included in systematic review and meta-analysis. Abbreviation: RCTs, randomized controlled trials

The scheme of literature search is shown in Fig. 1. In total, 4135 citations were originally selected through systematic review of electronic database. No other studies were included after title and abstract screening through manual search. Of these articles, 2174 were from PubMed, 1941 from Embase, and 20 from Cochrane library. After excluding duplicated documents, 3316 studies published before October 2022 remained. Further, we excluded 3273 studies that were identified to obviously not meet the inclusion criteria, after title and abstract screening. After fully reviewing 43 articles, 37 were excluded. Finally, 6 articles were included in this systematic review.

### Study characteristics

The baseline characteristics of the 6 enrolled studies are shown in Table 1 [2, 27–31]. The enrolled studies included 3 randomized controlled trials [2, 27, 31], 1 single center retrospective study [27], and 2 multicenter retrospective studies [28, 29]. The number of pediatric patients included in individual study ranged from 14 to 312. The reported mean or median age of patients ranged from 2.2 to 7.5 years. Saji et al. [30] reported the age range of the included patients (1 month to 17 years) instead of mean or median ages. The 6 included studies reported data on 287 patients administered with anti-inflammatory steroid treatments and 317 to conventional anti-failure therapy. Except for Aziz et al. [2] and Yang et al. [31], all other studies performed endomyocardial biopsy to diagnose acute myocarditis in the included patients. Other diagnosis methods including virus test, electrocardiography, echocardiography and chest radiography, were also used in all studies as supplements or complementary methods to confirm the disease progresses.

**Table 1:** Characteristics of included studies

Study	Yr	Type of study	Sample size	Study Duration	Age, Yrs	Treatment	Comparator
Aziz et al	2010	RCT	68	3 months	3.7	Prednisone 2 mg/kg/day for 15 days	Anti-failure medication
Camargo et al	1995	RCT	43	12 months	2.9 (Median)	Conventional therapy with various combinations of corticosteroids	Conventional therapy only—digitalis (Digoxin 10 Ixg/kg/day), diuretics (furosemide 1–4 mg/kg/day), and vasodilators (captopril 0.75–1.5 mg/kg/day)
English et al	2004	Single center retrospective study	41	N/A	2.2 (Median)	Steroid doses ranged from 2 to 10 mg/kg/day for a minimum of 3 days	N/A
Lin et al	2019	Multicenter retrospective study	312	N/A	2.9	Intravenous prednisolone used at > 10 mg/kg/day (steroids alone)	N/A
Saji et al	2012	Multicenter retrospective study	14	N/A	Between 1 month and 17 years	oral prednisolone (1–2 g/kg/day) or intravenous methylprednisolone pulse therapy	N/A
Yang et al	2006	RCT	132	3 months	7.5	Dexamethasone 0.2 mg/kg/day in week 1, 0.15 mg/kg/day in week 2, 0.075 mg/kg/day in week 3; together with ShenMai injection 0.4 ml/kg/day for 10 days	Conventional therapy (unclear)

*Abbreviations*: *RCT* Randomized clinical trial, *N/A* Not available

### Risk of bias in included studies

**Fig. 2:**
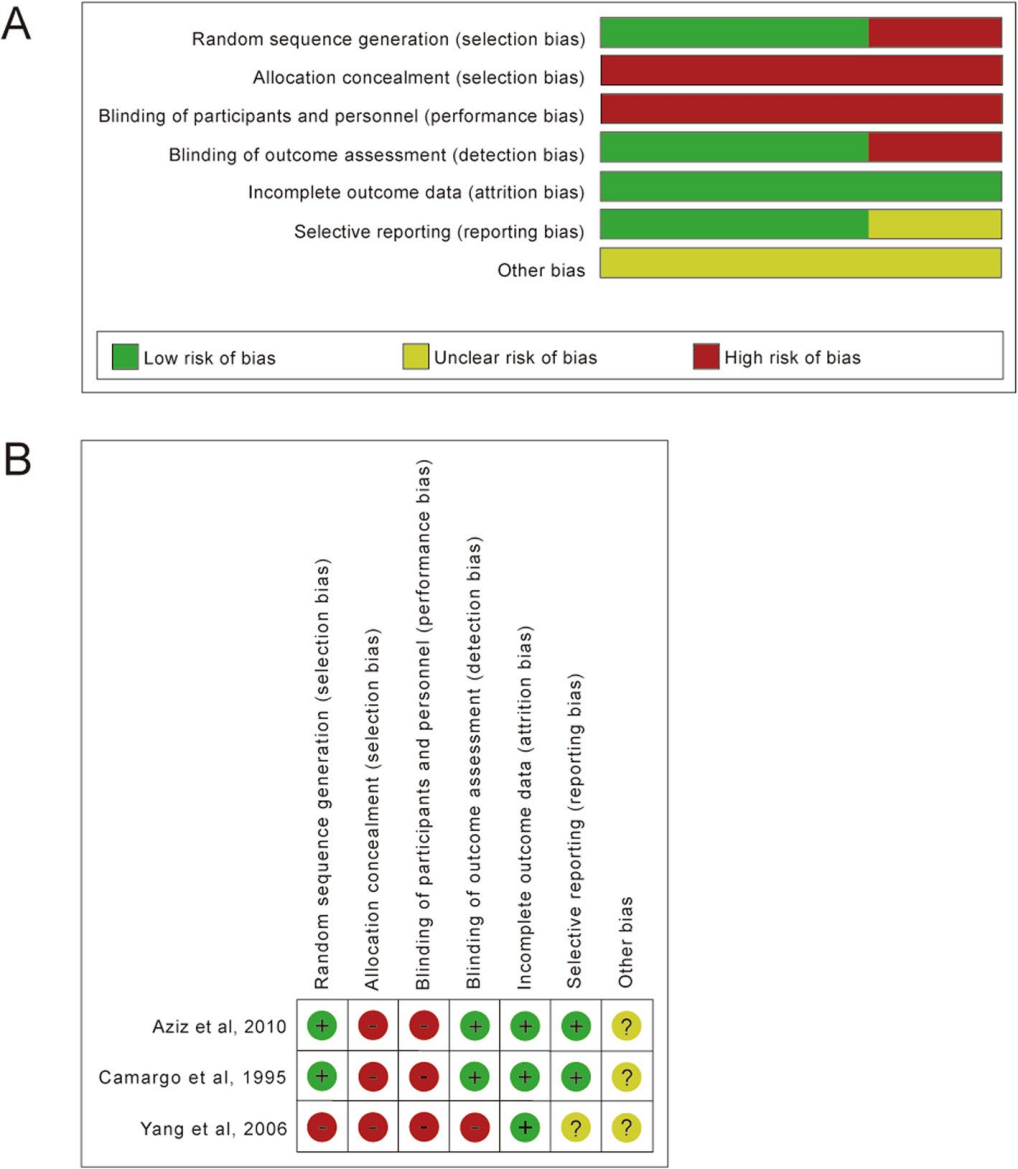
Risk of bias graph (**A**) and risk of bias summary (**B**) of all included RCTs about each risk of bias item presented as percentages across all included RCTs

The details of risk of bias in included RCTs are summarized in Fig. 2. Three included studies [28–30] are not randomized but retrospective studies, and are therefore not included in this analysis. Half of included studies [27, 28, 30] have small sample sizes less than 100 patients. None of the RCTs reported on allocation concealment, or blinding of participants and personnel.

### Efficacy of corticosteroid treatment on mortality due to acute myocarditis

**Fig. 3:**
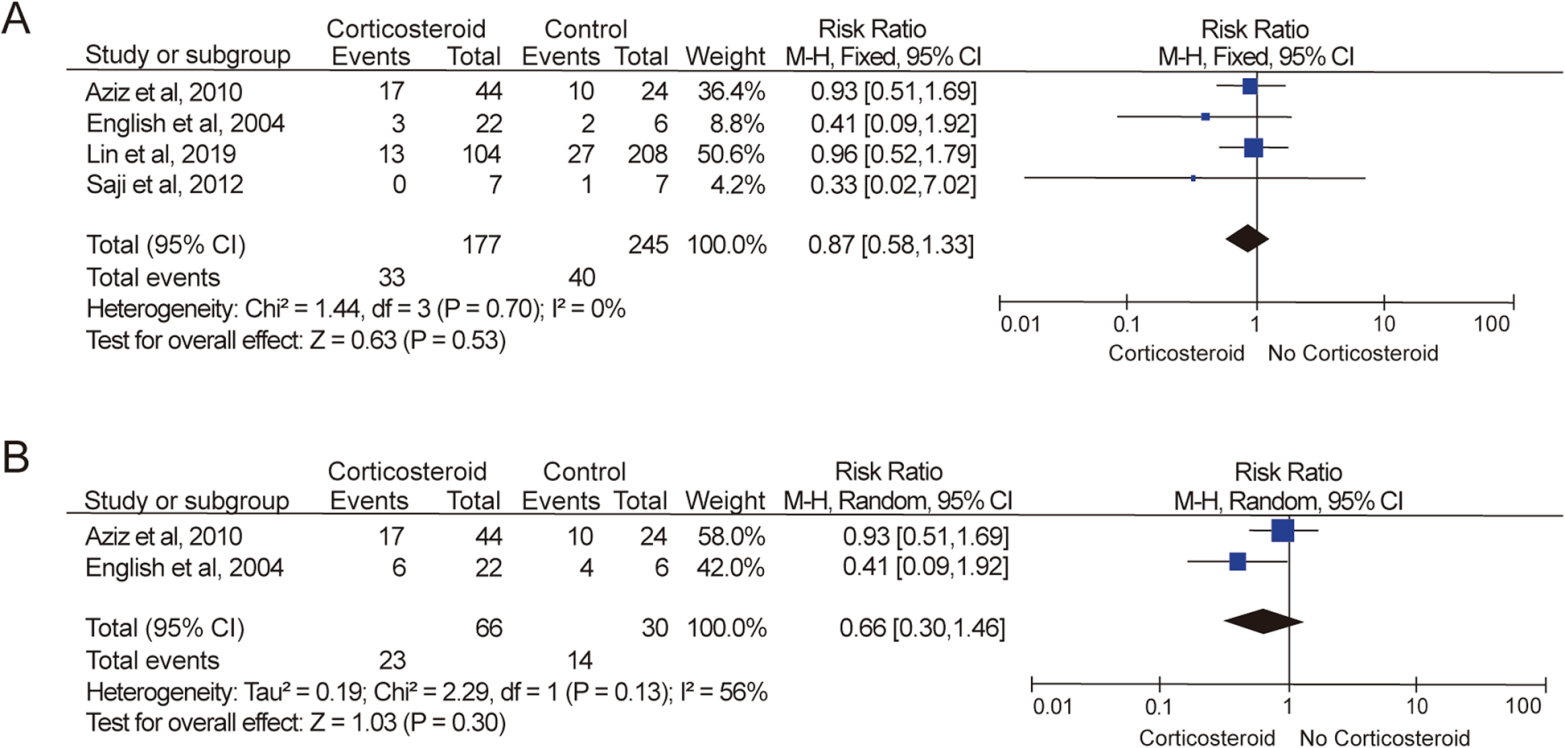
Assessment of mortality outcomes. **A** Meta-analysis of mortality in clinical trials and retrospective studies of corticosteroid anti-inflammatory therapy for pediatric acute myocarditis. **B** Meta-analysis of clinical endpoints (death or heart transplantation) in clinical trials and retrospective studies of corticosteroid anti-inflammatory therapy for pediatric acute myocarditis

Four studies [2, 28–30] reported mortality data for 422 pediatric patients. And in these 4 studies, 2 [2, 28] provided data for combination of death rate with heart transplantation rate. No statistically significant differences were observed between anti-inflammatory therapy and standard anti-failure treatment as shown in Fig. 3A. The data in these 4 studies indicated that there was no significant reduction in the risk of mortality for acute myocarditis in pediatric patients administered to corticosteroid treatment (*P* = 0.53; RR = 0.87; 95% CI = 0.58 to 1.33) based on the fixed-effect model (*I*^2^ = 0%, *P* = 0.70). In addition to the mortality rate data, Aziz et al. [2] and Camargo et al. [27] also provided evidence for the comparison of the combined outcome of mortality rate and heart transplantation rate in pediatric patients administered to corticosteroid treatment or conventional anti-failure therapies. As shown in Fig. 3B, high heterogeneity (*I*^2^ = 56%, *P* = 0.13) was seen in this meta-analysis which did not show statistically significant reduced risk of this clinical endpoint (*P* = 0.30; RR = 0.66; 95% CI = 0.30 to 1.46). However, this outcome was resulted from sharply different risk ratios in the two studies (0.93 and 0.41 for Aziz et al. [2] and Camargo et al. [27] respectively), indicating that no solid conclusions can be drawn from the current datasets.

### Efficacy of corticosteroid treatment on pediatric acute myocarditis patients’ left ventricular functions

**Fig. 4:**
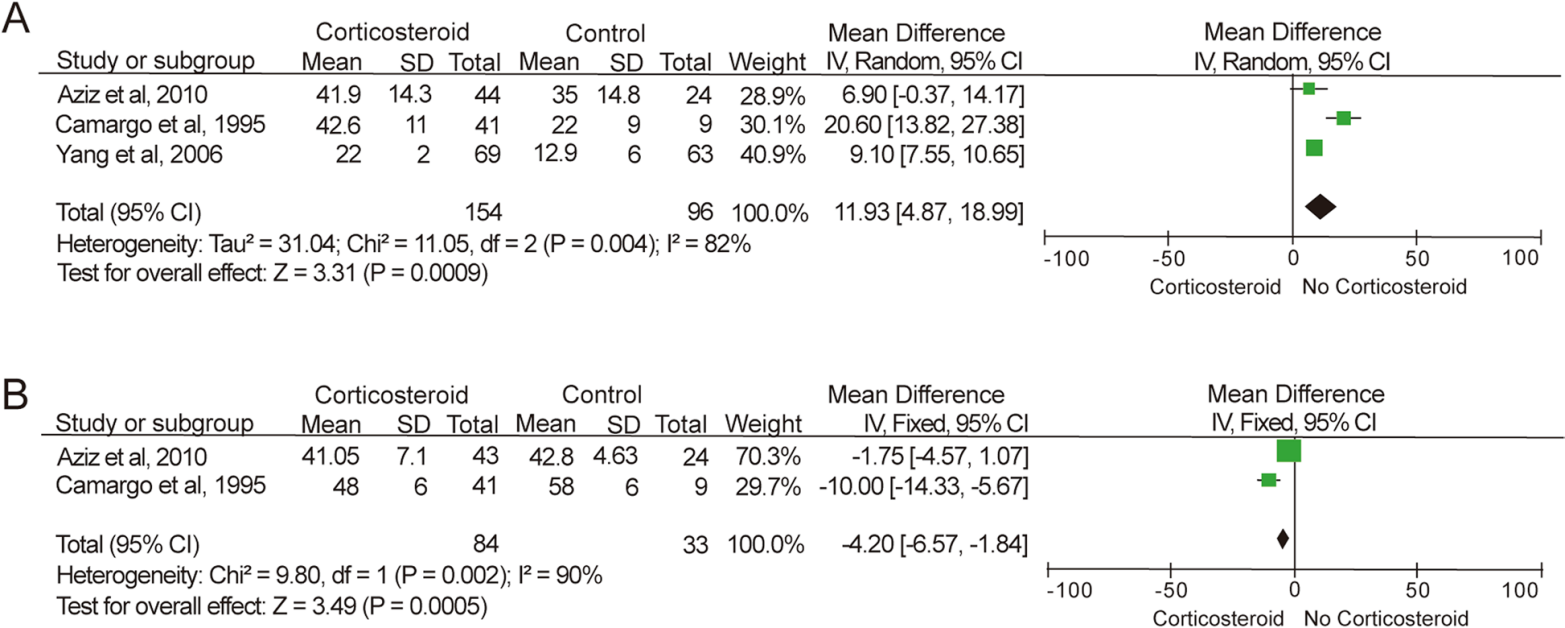
Assessment of left ventricular function. **A** Meta-analysis of LVEF in RCTs of corticosteroid anti-inflammatory therapy for pediatric acute myocarditis. **B** Meta-analysis of LVEDD in RCTs for pediatric acute myocarditis

The 3 RCTs [2, 27, 31] included in this paper reported on elevation of left ventricular compromises upon administration of steroid anti-inflammatory therapy. As shown in Fig. 4A, all the three studies reported outcome and changes in left ventricular ejection fraction (LVEF), while only Yang et al. [31] did not report changes in left ventricular end-diastolic diameter (LVEDD). In the comparison of LVEF measurements, significantly improvement was seen in pediatric patients randomized to steroid anti-inflammatory therapy compared to the control group (*P* = 0.0009; MD = 11.93%; 95% CI = 4.87% to 18.99%) based on the random-effect model (*I*^2^ = 82%, *P* = 0.004). The substantial heterogeneity can be reduced to 0% (*P* = 0.56) by removing the study conducted by Camargo et al. [27], while only slightly reducing the clinical improvement in LVEF from 11.93% to 9.00% (*P* < 0.0001; 95% CI = 7.48% to 10.52%). In term of the patients’ LVEDD at the end of observation, the data in studies conducted Aziz et al. [2] and Camargo et al. [27] had a substantial heterogeneity (*I*^2^ = 90%, *P* = 0.002) as shown in Fig. 4B. As the endpoint differences in LVEDD in the steroid treated pediatric patients and patients assigned to anti-failure treatment were sharply different in the two studies (-1.75 mm and -10.00 mm for Aziz et al. [2] and Camargo et al. [27] respectively), we cannot provide any conclusive argument on the assessment of effects of steroid anti-inflammatory treatment on LVEDD recovery in pediatric patients.

## Discussion

Cardiovascular disease is still a major cause of adverse outcomes in young individuals even though a unique, but still insufficiently characterized risk profile of cardiovascular disease presents in this group [32]. Among these diseases, the true incidence of acute myocarditis in pediatric patients is even more unpredictable owing to sub-clinical presentations estimated to be around 1 per 100,000 [33]. This number can still be undermined as pediatric acute myocarditis can be misdiagnosed initially as respiratory illness such as bronchiolitis or pneumonia, as myocarditis in children may not be presented with classic signs and symptoms [11]. The optimum treatment methods against pediatric acute myocarditis are still debatable, while the current treatment of myocarditis is still largely supportive. Corticosteroids, which are proposed to be immunosuppressive, have been widely used in immunological suppression therapeutics against myocarditis which has an autoimmune nature [23]. Although several different systematic reviews [23, 34, 35] have analyzed the role of corticosteroids in cardiovascular inflammatory disease treatment, the only systematic review focusing on pediatric acute myocarditis remained to be the one conducted by Hia et al. [17], a study conducted 20 years ago. As more clinical trials and retrospective studies in recent years [2, 28–31] provided us with more evidence of the clinical effectiveness of corticosteroid anti-inflammatory treatment of pediatric acute myocarditis, and thereby the basis of an updated analysis.

Our meta-analysis did not demonstrate a significant reduction in mortality rate of corticosteroid anti-inflammatory therapy compared to conventional anti-failure treatment without any other intervention (*P* = 0.53; RR = 0.87; 95% CI = 0.58 to 1.33). On the other hand, a significant enhancement of patients’ left ventricular function was identified in the meta-analysis of LVEF in the pediatric patient treated with corticosteroids compare to patients on supportive therapy (*P* = 0.0009; MD = 11.93%; 95% CI = 4.87% to 18.99%). Though high heterogeneity (*I*^2^ = 82%, *P* = 0.004) was observed with the inclusion of the study conducted by Camargo et al. [27], removal of this study from the meta-analysis still yielded a significant recovery in pediatric patients’ LVEF measurements (*P* < 0.0001; MD = 9.00%, 95% CI = 7.48% to 10.52%). Meanwhile, we also tried to conduct analysis on the clinical endpoint and LVEDD improvement in pediatric patients. However, in both meta-analyses, high heterogeneity was seen within 2 included studies. Therefore, we cannot draw any conclusion out of these meta-analyses.

As more and more recent reviews have pointed out that myocarditis is more common, and can be more severe in children than in adults [5, 7, 12], we think it is necessary to evaluate the immunosuppressive treatment of pediatric myocarditis rather than only relying on current data which mixed the clinical outcomes in populations of all ages. Two decades ago, when the public awareness on acute myocarditis in pediatric patients was still limited, Hia et al. [17] tentatively suggested that insufficient evidences can be used to conclude whether immunosuppressive therapy can lead to significantly improved outcomes in children with acute myocarditis. Since then, though Chen et al. [23] took a look at the effects of corticosteroids in general patients without a specific age range, not a single literature provided an insight on the effectiveness of anti-inflammatory therapy against acute myocarditis in pediatric patients. Unlike Hia et al. [17] which made a more qualitative measurement on improvement after immunosuppressive treatment, this systematic review is the first to provide insights on quantitative assessment of mortality and left ventricular function in children with acute myocarditis. At the same time, all the studies included in this systematic review are controlled studies which included at least 1 arm receiving only supportive treatment, while the majority of case studies [36–38] included in Hia et al. [17] had small sample sizes (no more than 10 patients), and lacked a control group, and thereby were likely to have biases in estimating the treatment effects.

Our study has several limitations. First, most of the studies included in this systematic review had small sample sizes (3 studies had a total sample size less than 50). The likelihood of overestimation of treatment effects is proportional to the percentage of small trials included in the meta-analysis. Adding the fact that not all included studies are RCTs, there could be potential issues with the methodological control of the available evidence. Second, in some of the meta-analyses performed in this study, significant heterogeneity was observed. The high heterogeneity, especially the comparison made for clinical endpoint after treatment, is highly likely due to the heterogeneous clinical features and patient background, such as the quality of care provided by the intensive care clinics in different countries and areas. Finally, none of the comparisons made in this study has included all trials and patients in this systematic review, as different studies provided different evidences to characterize the clinically important improvement of disease activity. This fact led to reduced sample sizes in all comparisons, and thereby increasing the difficulty of the assessment of bias in comparisons in which only 2 studies were included.

## Conclusions

This systematic review demonstrated no significant difference in mortality of pediatric patients with acute myocarditis with corticosteroid anti-inflammatory treatment. On the other hand, the administration of corticosteroid significantly improved the left ventricular function of pediatric patients with acute myocarditis based upon the measurement of LVEF at the endpoint of the clinical studies.

## Data Availability

The data sets analyzed during the current study are available from the corresponding author on reasonable request.
